# Adherence to Hydroxyurea and Patients’ Perceptions of Sickle Cell Disease and Hydroxyurea: A Cross-Sectional Study

**DOI:** 10.3390/medicina60010124

**Published:** 2024-01-10

**Authors:** Mohammed Ali Madkhali, Faisal Abusageah, Faisal Hakami, Basem Zogel, Khalid M. Hakami, Samar Alfaifi, Essam Alhazmi, Shaden Zaalah, Shadi Trabi, Abdulaziz H. Alhazmi, Mostafa Mohrag, Hafiz Malhan

**Affiliations:** 1 Department of Internal Medicine, Division of Hematology and Oncology, Faculty of Medicine, Jazan University, Jazan 45142, Saudi Arabia; 2 Faculty of Medicine, Jazan University, Jazan 45142, Saudi Arabia; abusageah.f.h@gmail.com (F.A.); faisalhakami1420@gmail.com (F.H.); basem14201@hotmail.com (B.Z.); hakamikhaled5@gmail.com (K.M.H.); samaralfaifi1@gmail.com (S.A.) e.alhazmi1420@gmail.com (E.A.); shdn1420@gmail.com (S.Z.); morooj11@hotmail.com (S.T.); abalhazmi@jazanu.edu.sa (A.H.A.); 3 Department of Medicine, Faculty of Medicine, Jazan University, Jazan 45142, Saudi Arabia; 4 Department of Hematology, Prince Mohammed Bin Nasser Hospital, Jazan 82943, Saudi Arabia; hmalhan@moh.gov.sa

**Keywords:** sickle cell disease, adherence, perception, hydroxyurea, Jazan, Saudi Arabia

## Abstract

*Background and Objectives*: Hydroxyurea is a crucial treatment for sickle cell disease (SCD), but some patients’ adherence to it remains suboptimal. Understanding patients’ perspectives on SCD and HU is essential for improving adherence. This study aimed to assess hydroxyurea adherence and patients’ perceptions of SCD and hydroxyurea among SCD patients in the Jazan region of Saudi Arabia. *Materials and Methods*: This cross-sectional study collected data from 217 SCD patients using self-administered questionnaires from August 2022 to January 2023. The survey covered patient demographics, SCD consequences, and other clinical data. We used the Brief Illness Perception Questionnaire (B-IPQ) to measure patients’ disease perception and the 8-item Morisky Medication Adherence Scale (MMAS-8) to evaluate patients’ adherence to HU. Data were analysed using descriptive, *t*-test, and chi-square tests, and the *p*-value was set at <0.05 for significance. *Results*: More than half of the patients were male, with a mean age of 28.09 ± 8.40 years. About 57.6% of the patients were currently using HU. About 81.6% of HU users reported low adherence. The adherence was lower among individuals with infections/recurrent infections and in patients who received repeated blood transfusions. ICU admission, blood transfusion, and certain SCD complications were associated with HU use. Male patients had a higher perception of SCD consequences, concern, and understanding. ICU-admitted and recurrent hospitalized patients had a higher perception of the SCD-related consequences, symptoms, concerns, and emotional responses. *Conclusions*: HU seems a well-established and efficacious disease-modifying agent, but its underutilization for SCD patients remains challenging. To overcome the adherence challenges, healthcare providers must educate SCD patients about the role of hydroxyurea in lowering disease severity and addressing side effects to obtain maximum benefits. Healthcare providers may consider tailored educational interventions to improve adherence, particularly for patients with infections, recurrent hospitalizations, or repeated blood transfusions. Further research is needed to identify strategies for improving hydroxyurea adherence and patient education among SCD patients.

## 1. Introduction

Sickle cell disease (SCD) belongs to a group of inherited disorders known as haemoglobinopathies, which are considered the most common inherited disorder affecting humans. SCD affects the developmental cycle of red blood cells (RBC), resulting in an abnormal form of haemoglobin (Hb) known as HbS [1,2]. The estimated prevalence of SCD is not well known as documented by the Centre for Disease Control and Prevention (CDC), but it affects millions of people worldwide, more commonly in Sub-Saharan Africa, Saudi Arabia, India, Mediterranean countries, and Central and South America [3]. In Saudi Arabia, the prevalence of SCD is estimated to be 24 per 10,000 people [4]. The eastern region has the highest prevalence of sickle cell disease and traits, which is 2.6% for SCD and 2% to 27% for the sickle cell trait (SCT), followed by the Jazan region [5,6].

Individuals with SCD are more likely to have intense painful episodes known as vaso-occlusive crises (VOC), which are the hallmark of the disease and occur when aberrant RBCs occlude small blood vessels [7]. Chronic haemolytic anaemia, pulmonary hypertension, acute chest syndrome (ACS), cerebrovascular accident (CVA), infections, hypoxia, and developmental impairment are all common consequences in those patients [7]. Due to the impact of disease-related consequences and frequent hospitalization, individuals with SCD suffer from a considerable reduction in all domains of the health-related quality of life (HRQoL) across their lifespan [8,9,10].

Throughout the last two decades, the treatment options for patients with SCD have been hydroxyurea (HU), blood transfusion, and stem cell transplantation [11,12]. Multiple pieces of evidence have shown that HU utilization in paediatric and adult patients significantly reduces morbidity and mortality and improves all domains of the HRQOL. Furthermore, it has been shown to be safe, cost-effective, and efficient for such patients [13,14].

Several barriers have been identified by The National Institutes of Health Consensus Development Statement on Hydroxyurea Treatment for SCD to ensure that patients are treated and benefit from HU, and one of the most important barriers is adherence to HU [15]. Patient-level limitations are thought to include a lack of understanding about HU, misconceptions about its detrimental consequences, and low health literacy [16]. Despite being approved as an SCD-modifying medication for decades, research has shown that HU is underutilized among adult SCD patients due to varying perceptions and beliefs [17]. Regarding parents’ perceptions, as reported, the majority of parents thought HU was a beneficial drug that encouraged their children to engage in greater physical activity, have fewer episodes of pain, and miss fewer days of school [18]. However, parental and patient views of treatment advantages versus disadvantages may depend on the severity of the disease and the benefits of HU [19].

The prevalence of SCD in the Jazan region is high, and there is a lack of data considering this topic in Jazan province. Therefore, the present study aimed to assess the adherence of patients with SCD to HU and to evaluate the factors that influence adherence in the Jazan region of Saudi Arabia, as well as how patients’ perceptions of SCD and HU affect their adherence; understanding patients’ and/or parents’ perceptions of SCD and HU is essential to creating behavioural interventions to enhance adherence.

## 2. Materials and Methods

We conducted this study using a cross-sectional observational design in Jazan Province, located in the southwest corner of Saudi Arabia [20]. We included patients of male and female sexes in Jazan Province who had already been diagnosed with SCD, who were 18 years of age or more, and who were presented to the reference hospital for haemoglobinopathies in the region, between August 2022 and January 2023. After obtaining ethical approval, a list of patients with SCD was provided, and the survey was sent to them. Those who were younger than 18 years of age, living outside Jazan province, and diagnosed with haemoglobin disorders other than SCD, and those who refused to participate, were excluded from the study. Data were gathered using an anonymous self-administrated questionnaire obtained from previously published studies [19,20,21]. The data were collected based on the networks of the trained data collectors and investigators of the study. The questionnaire was composed of three sections. The first section contained sociodemographic questions about matters such as age, sex, current education level of the patients, family income, history of various SCD complications, hospital or ICU admission, history of blood transfusion and surgical procedures, and associated comorbidities. The second section contained questions to assess patients’ awareness about HU and whether they were currently using it or not, followed by a brief illness perception questionnaire (B-IPQ) [22]. The B-IPQ is a tool used to assess a patient’s perception of their illness. It consists of eight items that measure the patient’s beliefs about their illness, including its consequences, personal control, treatment control, identity (symptoms), concern, understanding, and emotional representation. Each item is rated on a scale of 0–10, with higher scores indicating greater concern or negative perceptions about the illness. 

The last section involved the ©Modified Morisky Adherence Scale, 8-item (©MMAS-8) [23]. The ©MMAS-8 is a tool used to assess a patient’s HU adherence. It consists of eight items that measure the patient’s behaviour related to taking their HU over the past day and 2 weeks, including forgetfulness, carelessness, stopping medication when feeling better, and stopping medication when feeling worse. Each item is rated on a dichotomous scale (yes or no), with higher scores indicating better adherence. The patients were classified into three categories based on their scores: low adherence (0 to <6), medium adherence (6 to <8), and high adherence (8), respectively.

A pilot study was conducted before the distribution of the questionnaire, and it involved 20 patients to assess the clarity and wording of the questionnaire items and to correct any errors if present. Data obtained from this pilot study were excluded from the final analysis. 

We estimated that there were around 30,000 adult individuals with SCD in Jazan Province, and we used the Raosoft sample size calculator to determine the sample size for the study. We aimed to achieve a 95% confidence interval and 10% margin of error, with 50% prevalence of HU use among adults with SCD according to a previous study [24], which resulted in a sample size of 96 SCD patients. For the study, the convenience sampling technique was used. For the data that were gathered, descriptive statistics were presented. For statistical analysis, the one-way ANOVA test and independent t-test were also used. The 0.05 *p*-value cut-off was used in the statistical analysis of the data using SPSS v.25 (IBM Corp., Armonk, NY, USA). The study was authorized under the reference number No. 2278 on 11 August 2022 by Jazan Health Ethics Committee (REC) at the Ministry of Health. Before beginning the anonymous survey, each participant’s informed consent was requested and obtained. At any point during the research procedure, respondents had the option to abandon the survey. The participants’ anonymity and secrecy were preserved; no one was asked any questions that could have revealed their identity.

## 3. Results

### 3.1. Sociodemographic and Clinical Characteristics of the Study Participants

Table 1 summarizes the participants’ sociodemographic and clinical characteristics. A total of 217 patients with SCD met our inclusion criteria, and more than half of the patients were male, representing 54.4% of the study’ participants with a mean age of 28.09 ± 8.40 years (Table 1). About 87.1% of patients were hospitalized due to SCD consequences. Further, 88.9% of patients experienced at least one SCA painful crisis in the last three years. Furthermore, 27.6% of them had been admitted to the Intensive Care Unit (ICU). 

### 3.2. Hydroxyurea Use among the Participants and Its Association with the Sociodemographic and Clinical Characteristics of the Participants

Among all patients, more than half of them (125 patients) were currently using HU. A high monthly income was statistically associated with the increasing use of HU (*p*-value = 0.008). Additionally, those who had been previously admitted to the ICU, previously needed blood transfusion, or experienced complications like gallstone and joint necrosis were currently using HU more than those who had not been admitted to the ICU (*p*-value = 0.01, *p*-value = 0.001 and 0.038, respectively). 

### 3.3. Hydroxyurea Adherence among the Participants and Its Associated Factors

Regarding adherence to HU (Table 2), the patients were divided into two groups as low and moderate/high adherence. Of all the patients, 81.6% of them had low adherence to HU. The adherence rate was lower among those who had infections/recurrent infections and recurrent blood transfusions (*p*-value = 0.03 and 0.01, respectively). 

### 3.4. Perception of SCD/HU and Participants’ Characteristics, Healthcare Utilization, and Self-Reported Adherence

In our study, we examined the patient’s perception of SCD and HU in relation to the B-IPQ domains. The perception of SCD’s consequences, symptoms, and concern and its impact on the patient’ life was higher among the male sex (mean ± SD = 6.86 ± 2.98), with a statistically significant correlation (*p*-value = 0.003, 0.01, and 0.002, respectively). Patients with a lower adherence to HU had a higher perception of HU’s benefits in controlling the disease (mean ± SD = 5.77 ± 2.99), as shown in Table 3a.

Patients who had a history of ICU admissions had a higher perception of SCD’s consequences and symptoms (*p*-value = 0.016 and 0.001). Patients with more frequent hospitalizations perceived more about the SCD-related consequences (mean ± SD = 7.75 ± 2.92, *p*-value < 0.001), symptoms (mean ± SD = 7.62 ± 2.66, *p*-value < 0.001), concerns (mean ± SD = 7.44 ± 3.08, *p*-value = 0.001), and worse emotional responses to SCD (mean ± SD = 7.04 ± 3.13, *p*-value = 0.04), as described in Table 3c.

## 4. Discussion

SCD is one of the haemoglobinopathy disorders that has drawn major attention from public health due to its effects on increasing mortality and morbidity [25]. HU is a medication that has been found to lower the incidence and severity of painful crises and other SCD consequences [26]. Despite the high prevalence of SCD in Jazan province, there is a shortage of studies that assess the perceptions of HU and SCD and HU adherence. In this study, we found that the majority of participants were male, and more than half of the patients were on HU, but less than one-fifth of those patients were adherent to HU (Table 1 and Table 2). Using HU was associated with a high income, ICU admission, blood transfusion, and certain SCD complications (Table 1). Most HU users reported low adherence, particularly those with recurrent infections or frequent blood transfusions (Table 2). Male patients perceived more of SCD’s consequences and symptoms than females, while patients with more hospital admissions perceived more of SCD’s consequences, symptoms, concern, and emotional response (Table 3). 

Regarding the utilization of hydroxyurea in our study, we found that 125 (57.6%) of the SCD patients were currently using HU (Table 1). This finding was higher than what was reported in older studies published in Europe in 2008, 2016, and 2017, which reported that the proportion of patients on HU was 25%, 39%, and 40%, respectively [26,27,28]. In our region, in a study conducted in Oman in 2018, Jose et al. found that just 43% of the SCD patients were on HU [29]. Another study conducted in the Eastern region of Saudi Arabia in 2022 reported that 38% of the SCD patients were currently on HU [30]. Another study conducted in Riyadh, Saudi Arabia, from May 2017 to January 2018, revealed that approximately 78% of the patients were using HU [31]. However, in Jeddah, approximately 61% (in 2021) of the SCD patients were on HU [32]. These findings suggest that the utilization of HU among SCD patients varies widely between different regions and countries. 

Our findings suggest that the adherence to HU among SCD patients in Jazan Province, Saudi Arabia, is relatively low. In this study, 81.6% of the patients were found to have low adherence to HU, while only 18.4% had moderate or high adherence (Table 2). The adherence rates reported in this study are consistent with other studies conducted in the USA and Texas, specifically, which found that approximately 74% and 80% of the participants, respectively, had low adherence to HU [19,33]. However, a study conducted in England among paediatric patients reported higher adherence rates ranging from 77% to 85%. [18]. When it comes to the Middle East, a higher adherence rate to HU among SCD patients was reported in Oman, where 82% of the patients were found to have good adherence to HU [31]. According to a recent meta-analysis study, adherence rates were lower in older patients and those with more recognized barriers [34]; thus, the lower adherence in this study is noticeable as this study was conducted on participants with SCD who were 18 years of age or older. Moreover, it should be considered that low adherence rates in SCD patients may not be exclusive to HU, as the individuals’ beliefs influence the adherence-related behaviour regardless of the number of drugs prescribed, as found by Patel et al. in patients with SCD, wherein adherence to one medication was strongly associated with adherence to additional medications [35]. 

Our study found a significant association between the use of HU and various SCD complications, including ICU admission, blood transfusion, gallstones, and joint necrosis (Table 2). This finding contradicts a study conducted by Albohassan et al. in the Eastern Saudi Arabian province, which reported a decrease in painful crisis and the need for hospitalization with HU use [30]. This discrepancy in findings may be explained by the fact that most patients in our study were nonadherent to HU, and those with more severe SCD consequences were more likely to use HU compared to those with fewer consequences. Despite this, it is established that HU is effective in reducing severe painful episodes, hospitalizations, the number of blood transfusions, and acute chest syndrome and in improving survival [24,36]. Our study additionally found that adherence to HU was associated with a reduced risk of frequent blood transfusions and recurrent infections (Table 2). However, Namazzi et al. (2021) found that HU use reduced the incidence of clinically diagnosed infections, SCD consequences, and blood transfusions [37]. Thus, it may be considered that patients with a history of recurrent infections may be more likely to skip doses of HU due to disruptions in their daily routine or increased healthcare needs. 

The present study did not find a significant association between HU utilization and renal dysfunction, spleen removal, or pulmonary hypertension (Table 1). This lack of association was also observed when comparing these variables between patients who were adherent to HU and those who were not (Supplementary Materials Table S1). These findings are consistent with the BABY HUG randomized controlled trial, which reported no significant difference between HU and a placebo for the preservation of renal or splenic function as primary outcomes [29]. Similarly, in a cross-sectional study conducted by Gordeuk et al. in 2009 among patients with SCD, no significant change in the tricuspid regurgitant jet velocity, a widely used predictor of pulmonary hypertension, was observed between patients who were on HU and those who were not [38]. These studies provide further evidence that HU use is not significantly associated with renal dysfunction, spleen removal, or pulmonary hypertension in patients with SCD.

A study conducted in Chicago in 2017 by Badawy et al. found that participants with moderate or high adherence to HU reported experiencing more benefits of HU, fewer SCD symptoms, and a more positive emotional response compared to those with low adherence [19]. However, our study, which measured adherence to HU using the ©MMAS-8, did not find a significant association between adherence to HU and the perception of SCD according to the B-IPQ domains (Table 3a). Interestingly, low-adherence patients tended to perceive more control over their SCD treatment. Furthermore, several studies in other contexts have demonstrated a significant correlation between patients’ perceptions of the severity of their illness and adherence to their medications [39,40,41,42]. A noteworthy aspect of our study is that male patients perceived more of SCD’s consequences, symptoms, and concerns compared to female patients (Table 3(a)). This finding contradicts Badawy et al.’s previous study, which found that female patients perceived more SCD symptoms [19].

In our study, we found that SCD patients with more frequent hospitalization perceived more of SCD’s consequences, concerns, and symptoms, as well as a negative emotional response to SCD (Table 3c). Also, patients who had a history of ICU admissions perceived more consequences and symptoms of SCD (Table 3b). This result is consistent with the results from two studies conducted by Logan et al. and Mitchell et al. in 2002 and 2007, respectively, and they found that increased healthcare utilization was associated with parental perceptions of the severity of SCD and illness-related stress [43,44]. Similarly, an earlier study showed that patients’ perceptions of more SCD-related symptoms, consequences, worse emotional response to SCD, and a lower perceived benefit of HU were associated with increased hospitalizations and frequent emergency department visits [19]. However, patients and caregivers have different views on disease severity. Connelly et al. found that patients with SCD reported fewer symptoms and lower illness severity than their parents and physicians [45], but we did not investigate the SCD perceptions among various informants in our study, as we just enrolled adult patients with SCD in our study.

This study is one of the few to assess adherence to HU and patients’ perceptions of SCD and HU among adults in Jazan Province in Saudi Arabia, a country that bears a large burden of haemoglobinopathies. Thus, we believe this may evoke the need for enhanced programs to assure the better adherence of SCD patients to HU. This was a cross-sectional study using the convenience sampling method; so, it is to be anticipated that it has the limitations inherent to this method. The cross-sectional design limited our capacity to investigate changes in patients’ perceptions of SCD and adherence to HU over time. The effect of recall and selection bias cannot be disregarded because this study was conducted using an online survey. Those who did not participate because they lacked internet access or were unfamiliar with the platform may, therefore, have had an impact on the overall results. Future studies should consider the relationship between the duration of HU and the occurrence of VOC and other complications related to SCD. 

## 5. Conclusions

In SCD patients: perceptions of the disease and treatment were correlated with subjective and objective measures of adherence. Our findings showed that approximately 81% of patients using HU had low adherence to HU, and the adherence was lower among individuals with infections/recurrent infections and who had more blood transfusions. Regarding the perception of SCD/HU and participants’ characteristics, male patients perceived more of SCD’s consequences, concerns, and symptoms. ICU-admitted patients perceived more of SCD’s consequences and symptoms. Recurrently hospitalized patients perceived more SCD-related consequences, symptoms, concerns, and emotional responses. Adherence to HU is a multifactorial process, and alterations in patients’ perceptions of hydroxyurea help them to maintain a better disease control. For all these reasons, healthcare providers should consider patients’ beliefs about hydroxyurea when counselling about adherence at the time of the initial prescription and during follow-up visits with ongoing engagement in treatment decisions. In addition, initiatives to educate patients about the use, benefits, and potential side effects of hydroxyurea could help alleviate patients’ concerns about the drug and aid in overcoming adherence barriers. 

## Figures and Tables

**Table 1:** General sociodemographic, clinical characteristics, and hydroxyurea status among SCA patients in our study.

Sociodemographic Characteristics	Frequency (Percentage)	Hydroxyurea Status (Currently Use)		*p*-Value
*n* = 217		Yes 125 (57.6%)	No 92 (42.4%)	
Age (mean; SD)	28.09; 8.40	28.52; 8.14	27.50; 8.75	0.37
Gender				
Male	118 (54.4%)	63 (50.4%)	55 (59.8%)	0.17
Female	99 (45.6%)	62 (49.6%)	37 (40.2%)	
Family Monthly Income (in Riyals)				
Less than 5 k	57 (26.3%)	33 (26.4%)	24 (26.1%)	0.008 *
From 5 to 10 k	77 (35.5%)	53 (42.4%)	24 (26.1%)	
From 10 to 20 k	53 (24.4%)	20 (16%)	33 (35.9%)	
From 20 to 30 k	22 (10.1%)	15 (12%)	7 (7.6%)	
More than 30 k	8 (3.7%)	4 (3.2%)	4 (4.3%)	
Educational Level				
Uneducated	1 (0.5%)	1 (0.8%)	0 (0%)	0.93
Primary	6 (2.8%)	4 (3.2%)	2 (2.2%)	
Medium	6 (2.8%)	4 (3.2%)	2 (2.2%)	
Secondary	55 (25.3%)	31 (24.8%)	24 (26.1%)	
University	143(65.9%)	82 (65.6%)	61 (66.3%)	
Post-Graduate	6 (2.8%)	3 (2.4%)	3 (3.3%)	
How many times a year are you hospitalized for sickle cell anaemia episodes?				
0	28 (12.9%)	13 (10.4%)	15 (16.3%)	0.26
1	33 (15.2%)	20 (16%)	13 (14.1%)	
2	43 (19.8%)	23 (18.4%)	20 (21.7%)	
3	31 (14.3%)	20 (16%)	11 (12%)	
4	27 (12.4%)	12 (9.6%)	15 (16.3%)	
5	55 (25.3%)	37 (29.6%)	18 (19.6%)	
Number of episodes of pain crisis in the last three years				
0	24 (11.1%)	12 (9.6%)	12 (13%)	0.24
1	28 (12.9%)	16 (12.8%)	12 (13%)	
2	23 (10.6%)	10 (8%)	13 (14.1%)	
3	25 (11.5%)	11 (8.8%)	14 (15.2%)	
4	18 (8.3%)	12 (9.6%)	6 (6.5%)	
5	99 (45.6%)	64 (51.2%)	35 (38%)	
Number of episodes of acute chest syndrome in the last three years				
0	84 (38.7%)	47 (37.6%)	37 (40.2%)	0.32
1	35 (16.1%)	23 (18.4%)	12 (13%)	
2	37 (17.1%)	21 (16.8%)	16 (17%)	
3	19 (8.8%)	8 (6.4%)	11 (12%)	
4	17 (7.8%)	8 (6.4%)	9 (9.8%)	
5	25 (11.5%)	18 (14.4%)	7 (7.6%)	
Have you been admitted to the ICU?				
Yes	60 (27.6%)	43 (34.4%)	17 (18.5%)	0.01 *
No	157 (72.4%)	82 (65.6%)	75 (81.5%)	
Did you have an operation for spleen removal?				
Yes	15 (6.9%)	10 (8%)	5 (5.4%)	0.46
No	202 (93.1%)	115 (92%)	87 (94.6%)	
Did you receive blood transfusion due to SCD consequences?				
Yes	166 (76.5%)	104 (83.2%)	62 (67.4%)	0.007 *
No	51 (23.5%)	21 (16.8%)	30 (32.6%)	
Do you have other chronic diseases?				
Yes	175 (80.6%)	104 (83.2%)	71 (77.2%)	0.26
No	42 (19.4%)	21 (16.8%)	21 (22.8%)	
Gallstones				
Yes	98 (45.2%)	69 (55.2%)	29 (31.5%)	0.001 *
No	119 (54.8%)	56 (44.8%)	63 (68.5%)	
Pulmonary hypertension				
Yes	12 (5.5%)	7 (5.6%)	5 (5.4%)	0.95
No	205 (94.5%)	118 (94.4%)	87 (94.6%)	
Joint necrosis				
Yes	71 (32.7%)	48 (38.4%)	23 (25%)	0.038 *
No	146 (67.3%)	77 (61.6%)	69 (75%)	
Stroke				
Yes	17 (7.8%)	12 (9.6%)	5 (5.4%)	0.25
No	200 (92.2%)	113 (90.4%)	87 (94.6%)	
Kidney dysfunction				
Yes	14 (6.5%)	9 (7.2%)	5 (5.4%)	0.60
No	203 (93.5%)	116 (92.8%)	87 (94.6%)	
Recurrent infections or infections				
Yes	73 (33.6%)	46 (36.8%)	27 (29.3%)	0.25
No	144 (66.4%)	79 (63.2%)	65 (70.7%)	

SD: Standard deviation. ICU: Intensive care unit. * The alpha criterion for the *p*-value was set to 0.05.

**Table 2:** Self-reported adherence to hydroxyurea and its association with sociodemographic and clinical characteristics of the participants. (From Supplementary Material Table S1.)

Sociodemographic Characteristics	Hydroxyurea Adherence		*p*-Value
	Low	Moderate/High	
Frequency (*n* = 125)	102 (81.6%)	23 (18.4%)	
Age (mean; SD)	29.15; 8.43	25.74; 6.13	0.89
Did you do the blood transfusion because of the severe shortage of it?			
Yes	89 (87.3%)	15 (56.2%)	0.01 *
No	13 (12.7%)	8 (34.8%)	
Recurrent infections or infections			
Yes	42 (41.2%)	4 (17.4%)	0.03 *
No	60 (58.8%)	19 (82.6%)	

SD: Standard deviation. * The alpha criterion for the *p*-value was set to 0.05.

**Table 3:** **a**: Scores of B-IPQ according to the level of adherence to HU and gender. **b**: Scores of B-IPQ according to the history of blood transfusion for ICU admission. **c**: Scores of B-IPQ according to the number of hospital admission per year.

**(a)**											
**B-IPQ Domains**	**All (*n* = 217)**	**Hydroxyurea Adherence**					**Gender**				
		**Low**		**Moderate/High**		***p*-Value**	**Male**	**Female**		***p*-Value**	
Consequences	6.30; 3.08	6.46; 2.96		7.61; 2.5		0.08	6.86; 2.98	5.62; 3.07		0.003 *	
Personal Control	5.13; 3.05	5.32; 3.19		4.57; 2.67		0.29	4.82; 3.164	5.51; 2.88		0.10	
Treatment Control	5.77; 2.99	6.38; 2.89		4.78; 3.5		0.02 *	5.43; 3.04	6.18; 2.89		0.06	
Identity	6.26; 2.79	6.39; 2.66		6.65; 2.42		0.66	6.70; 2.79	5.74; 2.70		0.01 *	
Concerns	6.51; 3.04	6.42; 2.92		6.61; 3.05		0.78	7.10; 2.79	5.81; 3.18		0.002 *	
Understanding	7.92; 2.57	8.13; 2.38		8.09; 2.62		0.94	7.74; 2.66	8.14; 2.46		0.25	
Emotional Response	6.15; c2.26	5.99; 3.15		6.87; 2.98		0.22	6.40; 3.15	5.86; 3.75		0.25	
**(b)**											
**B-IPQ Domains**	**All (*n* = 217)**		**Blood Transfusion**				**ICU Admission**				
			**No**	**Yes**		***p*-Value**	**No**	**Yes**		***p*-Value**	
Consequences	6.30; 3.08		5.63; 3.17	6.51; 3.03		0.075	5.99; 2.98	7.12; 3.22		0.016 *	
Personal Control	5.13; 3.05		4.82; 3.04	5.23; 3.056		0.40	5.29; 2.885	4.73; 3.44		0.23	
Treatment Control	5.77; 2.99		5.51; 3.01	5.86; 2.98		0.47	5.85; 2.98	5.85; 3.01		0.56	
Identity	6.26; 2.79		5.67; 3.10	6.45; 2.67		0.08	5.86; 2.72	7.32; 2.71		0.001 *	
Concerns	6.51; 3.04		6.29; 3.28	6.58; 2.97		0.56	6.29; 2.99	7.08; 3.13		0.087	
Understanding	7.92; 2.57		7.43; 3.04	8.07; 2.40		0.12	7.83; 2.54	8.15; 2.65		0.42	
Emotional Response	6.15; 2.26		5.43; 3.34	6.37; 3.21		0.07	5.99; 3.31	6.58; 3.11		0.22	
**(c)**											
**B-IPQ Domains**	**All (*n* = 217)**	**Hospital Admission(s) per Year**									
		**0**		**1**	**2**	**3**	**4**		**5 or More**		***p*-Value**
Consequences	6.30; 3.08	4.54; 3.66		5.52; 3.04	5.98; 2.69	6.19; 2.57	6.78; 2.75		7.75; 2.93		<0.001 *
Personal Control	5.13; 3.05	5.79; 3.33		5.64; 2.21	5.53; 2.27	5.39; 2.66	4.67; 3.48		4.27; 3.21		0.14
Treatment Control	5.77; 2.99	5.68; 3.66		6.27; 2.30	5.63; 2.64	6.16; 3.43	5.93; 3.19		5.35; 2.97		0.74
Identity	6.26; 2.79	3.68; 3.019		5.42; 2.77	6.00; 2.04	6.74; 2.23	7.07; 2.36		7.62; 2.66		<0.001 *
Concerns	6.51; 3.04	4.54; 3.51		5.82; 2.68	6.65; 2.79	6.65; 2.81	7.15; 2.59		7.44; 3.08		0.001 *
Understanding	7.92; 2.57	7.36; 3.29		7.82; 2.40	7.91; 2.75	8.52; 2.42	8.15; 1.75		7.84; 2.91		0.65
Emotional Response	6.15; 2.26	5.11; 3.45		6.18; 3.08	5.67; 3.16	5.42; 3.48	7.00; 3.00		7.04; 3.13		0.04 *

* The alpha criterion for the *p*-value was set to 0.05

## Data Availability

The data presented in this study are available on request from the corresponding author.

## Supplementary Materials

The following are available online at https://www.mdpi.com/article/10.3390/medicina60010124/s1, Table S1: Self-reported adherence to hydroxyurea and its association with sociodemographic and clinical characteristics of the participants.
