# Supplementary material for: Adherence to Hydroxyurea and Patients’ Perceptions of Sickle Cell Disease and Hydroxyurea: A Cross-Sectional Study
Source: Medicina (Kaunas). 2024 Jan 10;60(1):124. doi: 10.3390/medicina60010124 (PMC10819561; doi:10.3390/medicina60010124)
Supplement: Supplementary file 1 [file medicina-60-00124-s001.zip › medicina-2702547-supplementary.pdf]

Table S1: self-report adherence to hydroxyurea and its association with sociodemographic and clinical characteristics of the participants.

Socio-demographic characteristics	Hydroxyurea Adherence		P-Value*
	Low	Moderate/High	
Frequency (n = 125)	102 (81.6%)	23 (18.4%)	
**Age (mean; SD)**	29.15; 8.43	25.74; 6.13	0.89
**Gender**			
Male	53 (52%)	10 (43.5%)	0.46
Female	49 (48%)	13 (56.6%)	
**Duration of the usage**			
Less than 1 year	11 (10.8%)	4 (17.4%)	0.37
1 year or more	91 (89.2%)	19 (82.6%)	
**The educational level of the mother**			
Uneducated	44 (43.1%)	10 (43.5%)	
High school diploma and below	34 (33.3%)	9 (39.1%)	0.507
Bachelor's degree	23 (22.5%)	3 (13%)	
Post-Graduate	1 (1%)	1 (4.3%)	
**The educational level of the father**			
Uneducated	14 (13.7%)	1 (4.3%)	
High school diploma and below	49 (48%)	15 (65.2%)	0.23
Bachelor's degree	35 (34.3%)	5 (21.7%)	
Post-Graduate	4 (3.9%)	2 (8.7%)	
**Family Monthly Income**			0.23
Less than 5 thousands	29 (28.4%)	4 (17.4%)	
From 5 to 10 thousands	41 (40.2%)	12 (52.2%)	
From 10 to 20 thousands	14 (13.7%)	6 (26.1%)	
From 20 to 30 thousands	14 (13.7%)	1 (4.3%)	

More than 30 thousands	4 (3.9%)	0 (0%)	
**Educational Level**			
Uneducated	1 (1%)	0 (0%)	0.934
primary	3 (2.9%)	1 (4.3%)	
Medium	3 (2.9%)	1 (4.3%)	
secondary	26 (25.5%)	5 (21.7%)	
University	66 (64.7%)	16 (69.6%)	
Post-Graduate	3 (2.9%)	0 (0%)	
**How many times a year are you hospitalized for sickle cell anemia episodes?**			
0 times	8 (7.8%)	5 (21.7%)	0.06
1 time	19 (18.6%)	1 (4.3%)	
2 times	20 (19.6%)	3 (13%)	
3 times	15 (14.7%)	5 (21.7%)	
4 times	12 (11.8%)	0 (0%)	
5 times or more	28 (27.5%)	9 (39.1%)	
**Number of episodes of pain crisis in the last three years**			
0 times	6 (5.9%)	6 (26.1%)	0.10
1 time	14 (13.7%)	2 (8.7%)	
2 times	9 (8.8%)	1 (4.3%)	
3 times	9 (8.8%)	2 (8.7%)	
4 times	10 (9.8%)	2 (8.7%)	
5 times or more	54 (52.9%)	10 (43.5%)	
**Number of episodes of acute chest syndrome in the last three years**			
0 times	35 (34.3%)	12 (52.2%)	0.46
1 time	18 (17.6%)	5 (21.7%)	
2 times	18 (17.6%)	3 (13%)	
3 times	8 (7.8%)	0 (0%)	

4 times	7 (6.9%)	1 (4.8%)	
5 times or more	16 (15.7%)	2 (8.7%)	
**Have you admitted to the ICU?**			0.35
Yes	37 (36.3%)	6 (21.1%)	
No	65 (63.7%)	17 (73.9%)	
**Did you have an operation for spleen removal?**			0.11
Yes	10 (9.8%)	0 (0%)	
No	92 (90.2%)	23 (100%)	
**Did you/did the blood transfusion because of the severe shortage of it?**			0.01*
Yes	89 (87.3%)	15 (56.2%)	
No	13 (12.7%)	8 (34.8%)	
**How many times have you had a broken limb (fracture)?**			0.86
0 times	75 (73.5%)	18 (73.3%)	
1 time	14 (13.7%)	3 (13%)	
2 times	5 (5.9%)	1 (4.3%)	
3 times	3 (2.9%)	0 (0%)	
4 times	3 (2.9%)	0 (0%)	
5 times or more	2 (2%)	1 (4.3%)	
**What's your blood type?**			0.36
O+	56 (54.9%)	13 (56.5%)	
O-	4 (3.9%)	0 (0%)	
A+	21 (20.6%)	4 (17.4%)	
A-	2 (2%)	1 (4.3%)	
B+	9 (8.8%)	1 (4.3%)	
B-	0 (0%)	0 (0%)	
AB+	1 (1%)	2 (8.7%)	
AB-	0 (%)	0 (0%)	
I don’t know	9 (8.8%)	2 (8.7%)	

**Gallstones**			
Yes	59 (57.8%)	10 (43.5%)	0.21
No	43 (42.2%)	13 (56.5%)	
**Pulmonary Hypertension**			
Yes	6 (5.9%)	1 (4.3%)	0.72
No	96 (94.1%)	22 (95.7%)	
**Joint Necrosis**			
Yes	40 (39.2%)	8 (34.8%)	0.63
No	62 (60.8%)	15 (65.2%)	
**Sores in the feet**			
Yes	13 (12.7%)	1 (4.3%)	0.24
No	89 (87.3%)	22 (95.7%)	
**Stroke**			
Yes	9 (8.8%)	3 (13%)	0.53
No	93 (91.2%)	20 (87%)	
**Kidney dysfunction**			
Yes	6 (5.9%)	3 (13%)	0.23
No	96 (94.1%)	20 (87%)	
**Vision problems**			
Yes	36 (35.3%)	7 (30.4%)	0.65
No	66 (64.7%)	16 (69.6%)	
**Recurrent infections or infections**			
Yes	42 (41.2%)	4 (17.4%)	0.03*
No	60 (58.8%)	19 (82.6%)	
**Do you have other chronic diseases?**			
Yes	83 (81.4%)	21 (91.3%)	0.25
No	19 (18.6%)	2 (8.7%)	
